# Association of Visceral Obesity Indices With Incident Diabetic Retinopathy in Patients With Diabetes: Prospective Cohort Study

**DOI:** 10.2196/48120

**Published:** 2024-02-06

**Authors:** Jiaheng Chen, Yu Ting Li, Zimin Niu, Zhanpeng He, Yao Jie Xie, Jose Hernandez, Wenyong Huang, Harry H X Wang, Guangzhou Diabetic Eye Study Group

**Affiliations:** 1 School of Public Health Sun Yat-Sen University Guangzhou China; 2 State Key Laboratory of Ophthalmology, Zhongshan Ophthalmic Center Sun Yat-Sen University Guangzhou China; 3 Guangdong Provincial Key Laboratory of Ophthalmology and Visual Science, Zhongshan Ophthalmic Center Sun Yat-Sen University Guangzhou China; 4 Liwan Central Hospital of Guangzhou Guangzhou China; 5 School of Nursing The Hong Kong Polytechnic University Hung Hom, Kowloon China (Hong Kong); 6 Faculty of Medicine and Health EDU, Digital Education Holdings Ltd Kalkara Malta; 7 Green Templeton College University of Oxford Oxford United Kingdom; 8 JC School of Public Health and Primary Care, Faculty of Medicine The Chinese University of Hong Kong Shatin, New Territories China (Hong Kong); 9 Usher Institute, Deanery of Molecular, Genetic & Population Health Sciences The University of Edinburgh Edinburgh United Kingdom

**Keywords:** Chinese visceral adiposity index, community-based cohort, diabetic retinopathy, lipid accumulation product, visceral adiposity index, visceral obesity indices

## Abstract

**Background:**

Visceral adipose tissue plays an active role in the pathogenesis of type 2 diabetes and vascular dysfunction. The lipid accumulation product (LAP), visceral adiposity index (VAI), and Chinese VAI (CVAI) have been proposed as simple and validated surrogate indices for measuring visceral adipose tissue. However, the evidence from prospective studies on the associations between these novel indices of visceral obesity and diabetic retinopathy (DR) remains scant.

**Objective:**

This study aimed to investigate the longitudinal associations of LAP, VAI, and CVAI with incident DR in Chinese patients with diabetes.

**Methods:**

This was a prospective cohort study conducted in Guangzhou in southern China. We collected baseline data between November 2017 and July 2020, while on-site follow-up visits were conducted annually until January 2022. The study participants consisted of 1403 patients with a clinical diagnosis of diabetes, referred from primary care, who were free of DR at baseline. The LAP, VAI, and CVAI levels were calculated by sex-specific equations based on anthropometric and biochemical parameters. DR was assessed using 7-field color stereoscopic fundus photographs and graded according to the modified Airlie House Classification scheme. Time-dependent Cox proportional hazard models were constructed to estimate the hazard ratios with 95% CIs. Restricted cubic spline curves were fitted to examine the dose-response relationship between the 3 indices of visceral obesity and new-onset DR. Subgroup analyses were performed to investigate the potential effect modifiers.

**Results:**

The mean age of study participants was 64.5 (SD 7.6) years, and over half (816/1403, 58.2%) were female. During a median follow-up of 2.13 years, 406 DR events were observed. A 1-SD increment in LAP, VAI, or CVAI was consistently associated with increased risk for new-onset DR, with a multivariable‑adjusted hazard ratio of 1.24 (95% CI 1.09-1.41; *P*=.001), 1.22 (95% CI 1.09-1.36; *P*<.001), and 1.48 (95% CI 1.19-1.85; *P*=.001), respectively. Similar patterns were observed across tertiles in LAP (*P* for trend=.001), VAI (*P* for trend<.001), and CVAI (*P* for trend=.009). Patients in the highest tertile of LAP, VAI, and CVAI had an 84%, 86%, and 82% higher hazard of DR, respectively, compared to those in the lowest tertile. A nonlinear dose-response relationship with incident DR was noted for LAP and VAI (both *P* for nonlinearity<.05), but not for CVAI (*P* for nonlinearity=.51). We did not detect the presence of effect modification by age, sex, duration of diabetes, BMI, or comorbidity (all *P* for interaction>.10).

**Conclusions:**

Visceral obesity, as measured by LAP, VAI, or CVAI, is independently associated with increased risk for new-onset DR in Chinese patients with diabetes. Our findings may suggest the necessity of incorporating regular monitoring of visceral obesity indices into routine clinical practice to enhance population-based prevention for DR.

## Introduction

Diabetes is an important global public health priority, with an estimated adult prevalence of 10.5% in 2021, rising to 12.2% in 2045 worldwide [1,2]. It poses an enormous threat to health and health care due to the associated mortality, disability, and costly long-term complications [3-5]. As one of the most common microvascular complications, diabetic retinopathy (DR) affects more than one-third of patients with diabetes and remains the leading cause of preventable visual impairment and blindness in working-age adults [6-9]. Given the growing prevalence of diabetes on a global scale [2,10], people at risk for DR are projected to increase rapidly over the coming decades [11]. Identifying modifiable risk factors for DR becomes critically imperative to inform clinical practice and public health recommendations in the context of addressing the ongoing epidemic of DR [12,13].

Observational epidemiologic studies have suggested hyperglycemia and hypertension as major risk factors for DR [6,14]; whereas clinical trial data demonstrated that stand-alone intensive control of blood glucose and blood pressure (BP) might not suffice to significantly reduce DR risk [15]. Meanwhile, genetic data show that the development of type 2 diabetes and obesity share environmental exposures and mechanisms [16]. Despite the established relationship between obesity and type 2 diabetes [17,18], meta-analyses of the association between overweight or obesity and DR have yielded mixed results [19-21]. Recent studies have reported that the distribution of adipose tissue rather than the total amount of fat is more important in the pathogenesis of insulin resistance, diabetes, and vascular dysfunction [22-25], thereby suggesting that visceral adipose tissue (VAT), in contrast to subcutaneous adipose tissue (SAT), tends to play a greater role in the development of DR. Nevertheless, previous studies mainly focused on generalized adiposity (as defined by the BMI) [26] or simple abdominal adiposity (as defined by waist-to-hip ratio [WHR]) [27,28], instead of more specific visceral adiposity.

At present, computed tomography (CT) and magnetic resonance imaging (MRI) techniques have been regarded as the “gold standard” for direct measurement of VAT [29]. Although the imaging technique has been used in large-scale studies in the West, such as the UK Biobank [30], it is less likely to be routinely adopted owing to its expensiveness and complex procedures [31]. Dual-energy x-ray absorptiometry (DEXA) and bioelectrical impedance analysis (BIA) have been used alternatively in epidemiological surveys [32,33]. However, access to these modalities to assess VAT, on top of the routine measures in clinical practice, may require trained technicians and dedicated facilities, with additional workload on health care in low-resource settings [29].

The lipid accumulation product (LAP), visceral adiposity index (VAI), and Chinese visceral adiposity index (CVAI), which could be easily implemented, have therefore been proposed as surrogate indices of VAT for wider use. The validation of these novel indices, when compared to traditional anthropometric adiposity measures, has demonstrated higher accuracy of visceral obesity discrimination and better prediction of type 2 diabetes [34-38]. However, findings from existing studies, mostly using a cross-sectional design, on associations between these indices of visceral obesity and DR are largely inconsistent [39-41]. More evidence from prospective studies is required to address this area of controversy. Therefore, we aimed to investigate the longitudinal associations of 3 validated visceral obesity indices, that is, LAP, VAI, and CVAI, with incident DR in patients with diabetes.

## Methods

### Study Design and Participants

This is an ongoing prospective cohort study among Chinese patients with diabetes conducted in Guangzhou in southern China. The study design was reported in detail elsewhere [28,42]. In brief, the participants consisted of primary care patients aged between 30 and 80 years with a clinical diagnosis of type 2 diabetes. All participants were referred through a generalist-specialist alliance consisting of 18 community health centers to a national-leading tertiary hospital specializing in ophthalmology (Zhongshan Ophthalmic Center), where a dilated, comprehensive eye examination was provided free of charge at baseline and at annual follow-up visits. The presence of type 2 diabetes was assessed by the attending primary care physician according to the Chinese Diabetes Society guideline and the World Health Organization recommendation when fasting plasma glucose ≥7.0 mmol/L; 2-hour plasma glucose ≥11.1 mmol/L during a 75-g oral glucose tolerance test; or hemoglobin A_1c_ (HbA_1c_) ≥6.5% [43,44]. All participants had their HbA_1c_ tested at the Zhongshan Ophthalmic Center to ensure that all enrolled patients who were not on glucose-lowering medication met the diagnostic criteria for diabetes. We excluded patients with type 1 (insulin-dependent) diabetes or female patients with clinically diagnosed gestational diabetes. The inclusion and exclusion criteria were described in detail in Multimedia Appendix 1 [35,36,45-51].

A total of 2975 patients with diabetes were enrolled between November 2017 and July 2020 and were followed up until January 2022. We excluded patients diagnosed with DR (n=803) and those with missing information on DR or visceral obesity (n=79) at baseline. Patients who were lost to follow-up (n=566) or without information on incident DR (n=124) were also excluded (Figure S1 in Multimedia Appendix 1). The final analysis included 1403 patients, most of whom attended 3 follow-up visits. A standardized examination procedure was performed at each visit.

### Data Collection

Information on sociodemographic characteristics (ie, sex, age, and education level), lifestyle behaviors (ie, smoking and drinking status), medical history (ie, duration of diabetes and the presence of comorbidities), and medication use (ie, glucose-lowering agents, antihypertensive medications, and lipid-lowering drugs) was collected through a face-to-face questionnaire administered by trained clinical staff. Anthropometric and biochemical measurements, with a venous blood sample taken, were performed following routine clinical procedures at the Zhongshan Ophthalmic Center.

Height and weight were measured with the patient in the standing position, wearing light clothing but not shoes, by a calibrated digital scale (HNH-318, Omron), to the nearest 0.1 cm and 0.1 kg, respectively. BMI was calculated as weight in kilograms divided by height in meters squared (kg/m^2^). Waist circumference (WC) was taken at the midpoint between the lower margin of the last palpable rib and the top of the iliac crest, while hip circumference was measured at the largest circumference around the buttocks, both to the nearest 0.1 cm. Waist-to-height ratio (WHtR) was calculated as WC (cm) divided by height (cm). The WHR was calculated as WC (cm) divided by hip circumference (cm). BP was measured in a seated position after a 10-minute rest by routinely validated automatic sphygmomanometers (HEM-907, Omron). The arm with the higher BP values was used. The average of 2 BP readings, 1-2 minutes apart, was recorded. Serum creatinine and lipid profiles, including plasma cholesterol and triglycerides, were directly measured using an automatic biochemistry modular analyzer (Cobas 8000, Roche Diagnostics). HbA_1c_ was measured by an automated, high-performance liquid chromatography system (G8, Sysmex Corporation). The estimated glomerular filtration rate (eGFR) was calculated using the Chronic Kidney Disease Epidemiology Collaboration equation for Asians [52]. Detailed information on the definition and categorization of variables is provided in Multimedia Appendix 1.

### Assessment of Visceral Obesity Indices

Visceral obesity was assessed using 3 validated indices, that is, LAP, VAI, and CVAI, all of which have demonstrated satisfactory correlations with VAT measured using CT scans [34,36]. The indices were calculated based on demographics (sex and age), anthropometrics (BMI and WC), and metabolic parameters (triglycerides and high-density lipoprotein cholesterol). The detailed formulas were described in Multimedia Appendix 1. Given the lack of consensus regarding the optimal cutoff point for identifying visceral obesity and the considerable disparities in the amount of VAT between males and females, the 3 indices were divided into sex-specific tertiles when treated as categorical variables.

### Fundus Examination and Grading of DR

All study participants had color stereoscopic fundus photographs of seven standard fields taken in each eye after pupil dilation using a digital retinal camera (Canon CR-2). The photographs were graded at the Zhongshan Ophthalmic Center using the modified Airlie House Classification scheme as adapted for the Early Treatment Diabetic Retinopathy Study (ETDRS) [53]. A total of 2 trained ophthalmic specialists independently graded the fundus photographs, with disagreements (<8%) being resolved by the decision of a senior ophthalmologist. The grading of DR was determined based on the worst eye, ranging from the ETDRS classification levels from 10 to 85, that is, levels 10-20 (no apparent DR), level 35 (mild nonproliferative DR [NPDR]), levels 43-47 (moderate NPDR), level 53 (severe NPDR), and levels 61-85 (proliferative DR) [53]. Incident DR was defined as eyes with no apparent DR at baseline in which any DR was newly diagnosed at follow-up.

A more precise evaluation of retinal pathology in diabetic macular edema (DME) was performed with a swept-source optical coherence tomography device (DRI-OCT Triton, Topcon). The swept-source optical coherence tomography volume was captured in a 3D scan pattern over a 6×6 mm area for all eyes. The presence of DME, which was assessed separately from that of DR, was characterized by retinal thickening or hard exudates in the posterior pole that can develop at any stage of DR [54]. Given that the vision-threatening DR (VTDR) includes severe NPDR, proliferative DR, or DME [55,56], the presence of DME was taken into account in the sensitivity analysis.

### Statistical Analysis

The Pearson correlation coefficient was examined for the 3 visceral obesity indices (ie, LAP, VAI, and CVAI) and the conventional obesity measures (ie, BMI, WC, WHtR, and WHR), respectively. Patients enrolled at baseline were followed until they were diagnosed with any DR or the recorded attendance at the most recent follow-up visits until January 2022, whichever came first. The incidence density was reported as the number of outcome events per 100 person-years. Time-dependent Cox proportional hazard models were constructed to explore the associations of LAP, VAI, and CVAI with incident DR at follow-up. The 3 visceral obesity indices and all other covariates, except for sex and education level, were considered time-varying variables in the main analysis. The model construction was described in detail in Multimedia Appendix 1.

Hazard ratios (HRs) with 95% CIs were estimated from the crude and adjusted models. Model 1 was adjusted for sex, age, and duration of diabetes. Model 2 was further adjusted for education level, current smoking, regular drinking, BMI, BP, HbA_1c_, low-density lipoprotein cholesterol, eGFR, and use of insulin. Variable selection was determined from our previous knowledge of sociodemographic factors, health-related lifestyles, and anthropometric and biochemical parameters [6,9,14,55]. The proportional hazards assumption for model fit was tested using the scaled Schoenfeld residuals. The variance inflation factors were examined to ensure the absence of multicollinearity (ie, all variance inflation factors <5) in the regression model. We further modeled data as restricted cubic splines (RCSs) with 4 knots, located at the 5th, 35th, 65th, and 95th percentiles following the Akaike information criterion [57] of LAP, VAI, and CVAI, respectively, to assess the shape of the association between the 3 visceral obesity indices and risk of DR. We performed separate Cox regression analyses across patient subgroups according to sex (female vs male); age (<65 vs ≥65 years old); duration of diabetes (<10 vs ≥10 years); BMI (<24 vs ≥24 kg/m^2^); and the presence of concurrent medical conditions including hypertension (yes vs no), dyslipidemia (yes vs no), and decreased renal function (yes vs no) to investigate the potential effect modifiers. The interaction between visceral obesity and the stratifying variable was explored by inserting a 2-factor interaction term into the regression model.

A series of sensitivity analyses were performed. First, we added the presence of DME to the outcome of interest, which was redefined as new-onset DR or DME at follow-up in patients free of both DR and DME at baseline. Second, we excluded patients who had incident DR within the first year of follow-up to account for the possible reverse causality bias. Third, we estimated HR from the Cox regression models without adjustment for BMI to avoid the plausible bias associated with over-adjustment. Fourth, we incorporated WC as a covariate in the regression model to detect whether the visceral obesity indices may have a role independent of abdominal obesity. We also estimated HR from time-fixed Cox regression models, in which baseline values of LAP, VAI, and CVAI were used to ascertain whether associations between visceral obesity indices and incident DR may vary from the time-dependent main analysis. Analyses were conducted using Stata (version 15.1; StataCorp LLC) and R (version 4.2.2; Core Team). A 2-tailed *P*<.05 was considered statistically significant.

### Ethical Considerations

Data anonymization was performed by removing all patient identifiers from the data set before data analysis. Ethics approval was granted by the Zhongshan Ophthalmic Center Medical Ethics Committee (2017KYPJ094) at Sun Yat-Sen University as per the Declaration of Helsinki 2013. All participants provided written, informed consent.

## Results

### Characteristics of Study Participants

Of the 1403 patients included in the final analysis, slightly over half (816/1403, 58.2%) were female. The mean (SD) age of patients was 64.5 (7.6) years at baseline. The median (IQR) values of LAP, VAI, and CVAI were 48 (29.12-77.22), 2.69 (1.54-4.43), and 118.18 (94.77-141.17), respectively. During a median follow-up period of 2.13 years, we documented 406 new-onset DR events (ie, 289 cases of mild NPDR, 116 cases of moderate NPDR, and 1 case of severe NPDR), with an incidence density of 15.71 per 100 person-years. Patients who experienced new-onset DR at follow-up tended to be female, younger, with a longer duration of diabetes, on insulin, and had higher HbA_1c_ and greater visceral obesity at baseline than their counterparts (Table 1). The correlation matrix showed statistically significant correlations among LAP, VAI, CVAI, BMI, WC, WHtR, and WHR (Table S1 in Multimedia Appendix 1). The sex distribution, duration of diabetes, lifestyle behaviors, comorbidity status, and levels of the majority of anthropometric and biochemical measurements were comparable between patients excluded during follow-up and those included in the final analysis, albeit slightly younger (64.45 vs 65.23 years) and with better glycemic control in the final sample (Table S2 in Multimedia Appendix 1).

**Table 1:** Baseline characteristics of study participants by incident diabetic retinopathy (DR) at follow-up. The 2-sample *t* test, Mann-Whitney U test, or chi-square test, where appropriate, was used for between-group comparison.

Characteristics		Absence of incident DR at follow-up (n=997)	Presence of incident DR at follow-up (n=406)	*P* value
Female sex, n (%)		560 (56.2)	256 (63.1)	.02
**Age (years)**				
	Overall, mean (SD)	64.72 (7.49)	63.77 (7.81)	.04
	≥65, n (%)	515 (51.7)	186 (45.8)	.047
**Duration of diabetes (years)**				
	Overall, median (IQR)	6.0 (3-11)	7.0 (3-13.5)	.002
	≥10, n (%)	344 (34.5)	162 (40)	.053
**Education level, n (%)**				.56
	Junior secondary school or below	316 (31.7)	120 (29.5)	
	Senior secondary school	399 (40)	175 (43.1)	
	College or above	282 (28.3)	111 (27.4)	
Current smoking, n (%)		130 (13)	55 (13.5)	.82
Regular drinking, n (%)		87 (8.7)	45 (11.1)	.20
Use of insulin, n (%)		140 (14)	93 (22.9)	<.001
**Presence of comorbidity, n (%)**				
	Hypertension	548 (55)	246 (60.6)	.054
	Dyslipidemia	662 (66.4)	276 (68)	.57
	Decreased renal function	414 (41.5)	163 (40.2)	.64
**BMI (kg/m²)**				
	Overall, mean (SD)	24.59 (3.26)	24.73 (3.29)	.47
	≥24, n (%)	552 (55.4)	229 (56.4)	.72
Waist circumference (cm), mean (SD)		85.87 (9.11)	86.17 (9.16)	.57
**Blood pressure (mm Hg), mean (SD)**				
	Systolic blood pressure	132.40 (17.98)	133.88 (18.45)	.17
	Diastolic blood pressure	70.02 (10.14)	71 (10.44)	.10
HbA_1c_^a^, (%), mean (SD)		6.71 (1.16)	7.04 (1.34)	<.001
**Cholesterol (mmol/L)**				
	Total cholesterol, mean (SD)	4.83 (1.09)	4.81 (0.98)	.72
	Triglycerides, median (IQR)	1.89 (1.31-2.85)	1.99 (1.39-2.93)	.21
	LDL-C^b^, mean (SD)	3.03 (0.99)	3.04 (0.85)	.89
	HDL-C^c^, mean (SD)	1.31 (0.42)	1.28 (0.36)	.14
Serum creatinine (μmol/L), mean (SD)		71.49 (19.68)	71.09 (19.66)	.73
eGFR^d^ (mL/min per 1.73 m^2^), mean (SD)		90.32 (16.91)	90.25 (16.92)	.95
**Indices, median (IQR)**				
	LAP^e^	46.55 (27.64-75.44)	51.70 (31.80-80.08)	.03
	VAI^f^	2.51 (1.47-4.37)	2.94 (1.79-4.54)	.003
	CVAI^g^	117.47 (93.28-140.07)	119.58 (96.39-144.91)	.12

^a^HbA_1c_: hemoglobin A1c.

^b^LDL-C: low-density lipoprotein cholesterol.

^c^HDL-C: high-density lipoprotein cholesterol.

^d^eGFR: estimated glomerular filtration rate.

^e^LAP: lipid accumulation product.

^f^VAI: visceral adiposity index.

^g^CVAI: Chinese visceral adiposity index.

### Association of Visceral Obesity Indices With Incident DR

**Figure 1:**
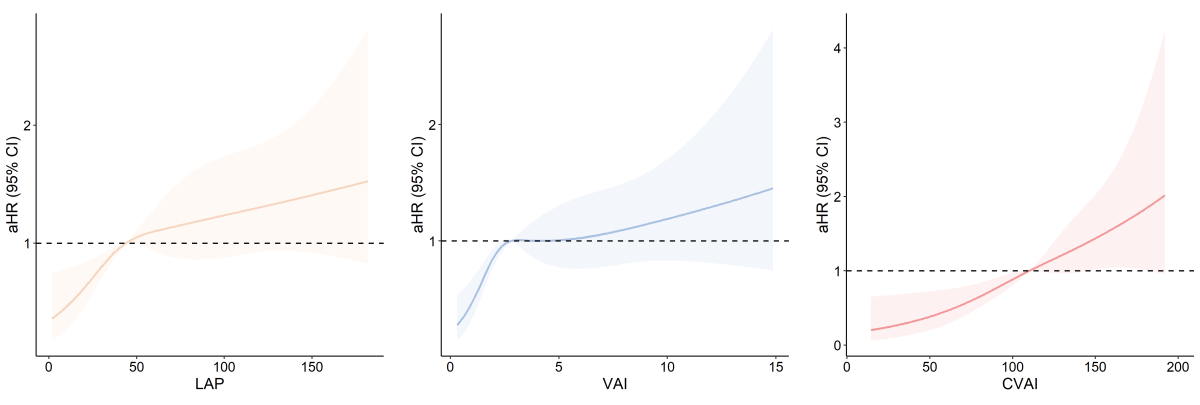
Dose-response relationships of lipid accumulation product (LAP), visceral adiposity index (VAI), and Chinese VAI (CVAI) with incident diabetic retinopathy. aHR: adjusted hazard ratio.

A 1-SD increment in LAP, VAI, or CVAI was consistently associated with increased risk for DR, with a multivariable adjusted HR (aHR) of 1.24 (95% CI 1.09-1.41; *P*=.001), 1.22 (95% CI 1.09-1.36; *P*<.001), and 1.48 (95% CI 1.19-1.85; *P*=.001), respectively (Table 2). Similar patterns were observed across tertiles in LAP (*P* for trend=.001), VAI (*P* for trend<.001), and CVAI (*P* for trend=.009). Patients in the highest tertile of LAP, VAI, and CVAI had an 84% (aHR=1.84; 95% CI 1.30-2.62), 86% (aHR=1.86; 95% CI 1.35-2.57), and 82% (aHR=1.82; 95% CI 1.16-2.86) higher hazard of new-onset DR, respectively, compared to those in the lowest tertile (Table 2). A positive, nonlinear dose-response relationship with incident DR was noted for LAP and VAI (both *P* for nonlinear trend<.05), but not for CVAI (*P* for nonlinear trend=.51; Figure 1).

**Table 2:** Time-dependent Cox proportional hazard models with and without adjustment for covariates. The crude model referred to time-dependent Cox proportional hazard models with no adjustment. Model 1 was adjusted for sex, age, and duration of diabetes. Model 2 was then further adjusted for education level, current smoking, regular drinking, BMI, blood pressure, hemoglobin A1c, serum cholesterol level, estimated glomerular filtration rate, and use of insulin.

Visceral obesity indices		Crude model		Model 1		Model 2	
		cHR^a^ (95% CI)	*P* value	aHR^b^ (95% CI)	*P* value	aHR (95% CI)	*P* value
**Lipid accumulation product^c^**							
	Per SD increase	1.12 (1.01-1.25)	.03	1.14 (1.03-1.27)	.02	1.24 (1.09-1.41)	.001
	Tertile 1	1.00 (Reference)	N/A^d^	1.00 (Reference)	N/A^d^	1.00 (Reference)	N/A^d^
	Tertile 2	1.19 (0.89-1.60)	.25	1.27 (0.93-1.72)	.13	1.31 (0.94-1.83)	.11
	Tertile 3	1.42 (1.07-1.89)	.02	1.53 (1.14-2.05)	.004	1.84 (1.30-2.62)	.001
	*P* for trend	N/A^d^	.02	N/A^d^	.005	N/A^d^	.001
**Visceral adiposity index^c^**							
	Per SD increase	1.18 (1.07-1.32)	.002	1.19 (1.07-1.32)	.001	1.22 (1.09-1.36)	<.001
	Tertile 1	1.00 (Reference)	N/A^d^	1.00 (Reference)	N/A^d^	1.00 (Reference)	N/A^d^
	Tertile 2	1.36 (1.01-1.84)	.04	1.45 (1.07-1.97)	.02	1.56 (1.13-2.17)	.007
	Tertile 3	1.60 (1.19-2.13)	.002	1.67 (1.24-2.24)	.001	1.86 (1.35-2.57)	<.001
	*P* for trend	N/A^d^	.003	N/A^d^	.001	N/A^d^	<.001
**Chinese visceral adiposity index^c^**							
	Per SD increase	1.16 (1.04-1.31)	.01	1.22 (1.07-1.37)	.002	1.48 (1.19-1.85)	.001
	Tertile 1	1.00 (Reference)	N/A^d^	1.00 (Reference)	N/A^d^	1.00 (Reference)	N/A^d^
	Tertile 2	1.25 (0.94-1.67)	.13	1.38 (1.02-1.86)	.04	1.44 (1.01-2.05)	.046
	Tertile 3	1.39 (1.04-1.85)	.03	1.53 (1.13-2.07)	.006	1.82 (1.16-2.86)	.009
	*P* for trend	N/A^d^	.03	N/A^d^	.006	N/A^d^	.009

^a^cHR: crude hazard ratio.

^b^aHR: adjusted hazard ratio.

^c^SD of lipid accumulation product=38.90, SD of visceral adiposity index=2.73, and SD of Chinese visceral adiposity index=34.23.

^d^N/A: not applicable.

Dose-response relationships were examined using RCS with 4 knots, located at the 5th, 35th, 65th, and 95th percentiles of LAP, VAI, and CVAI, respectively. The solid line represents the fitted curve, and the shaded areas represent the 95% CI bands. Time-dependent Cox proportional hazard models were adjusted for sex, age, duration of diabetes, education level, current smoking, regular drinking, BMI, BP, HbA_1c_, serum cholesterol level, eGFR, and use of insulin (test for overall trend: LAP *P*=.001, VAI *P*<.001, and CVAI *P*=.006; test for nonlinear trend: LAP *P*=.048, VAI *P*=.002, and CVAI *P*=.51).

### Subgroup Analyses

**Figure 2:**
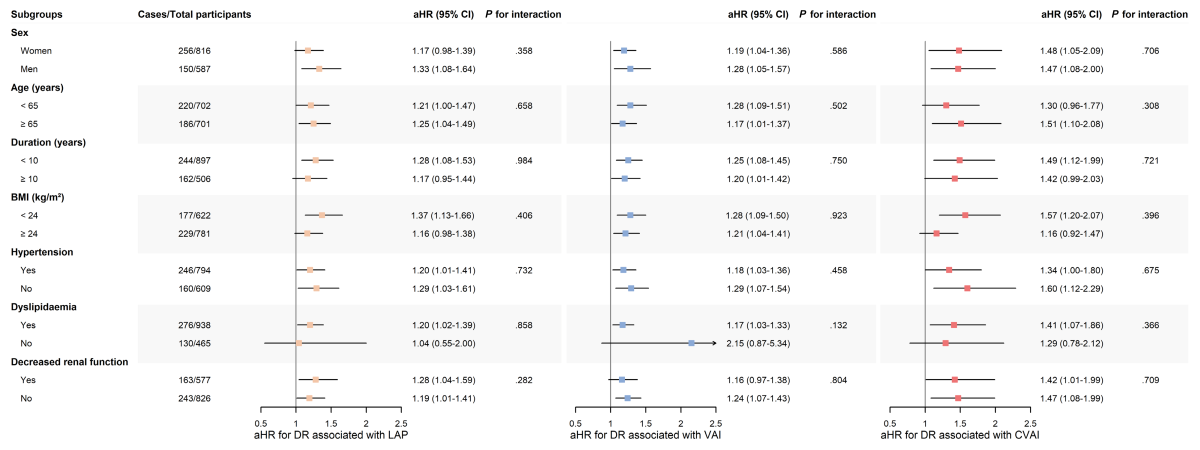
Associations of lipid accumulation product (LAP), visceral adiposity index (VAI), and Chinese VAI (CVAI) with incident diabetic retinopathy (DR) across patient subgroups. aHR: adjusted hazard ratio.

When patients were classified by sex, age, duration of diabetes, BMI, and the presence of concurrent hypertension, dyslipidemia, and decreased renal function, the associations of a 1-SD increment in LAP, VAI, and CVAI with new-onset DR observed in the main analysis remained consistent across all patient subgroups. Multivariable-adjusted Cox models, in which interactions between visceral obesity and the stratifying variables were explored, showed no evidence of effect modification by age, sex, duration of diabetes, BMI, or comorbidity (all *P* for interaction>.10; Figure 2).

Time-dependent Cox proportional hazard models were fitted across all patient subgroups, with each Cox model adjusted for sex, age, duration of diabetes, current smoking, regular drinking, BMI, BP, HbA_1c_, serum cholesterol level, eGFR, and use of insulin in all other subgroup analyses. The interaction between visceral obesity and the stratifying variable was explored by inserting a 2-factor interaction term into the regression model. The hazard of incident DR at follow-up associated with a 1-SD increment in LAP, VAI, or CVAI, respectively, was calculated.

### Sensitivity Analyses

**Figure 3:**
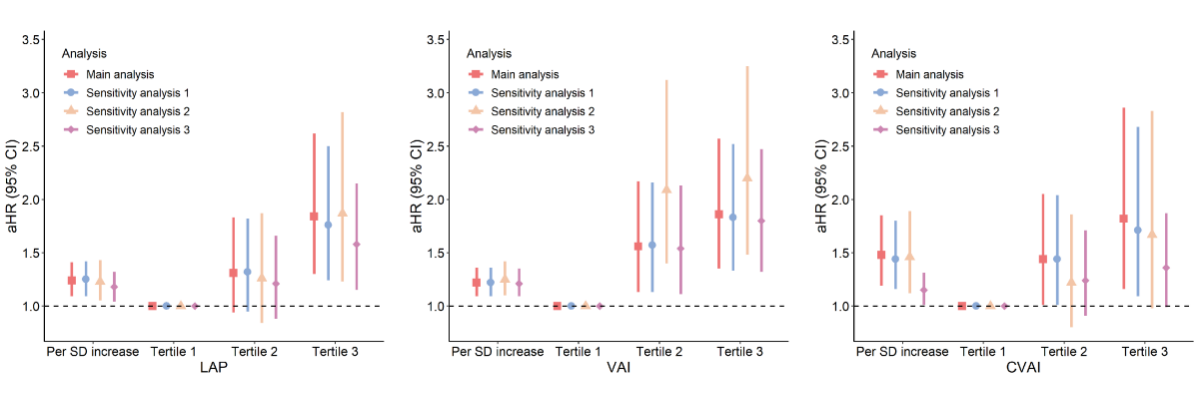
Associations of lipid accumulation product (LAP), visceral adiposity index (VAI), and Chinese VAI (CVAI) with incident diabetic retinopathy in the main analysis and sensitivity analyses. aHR: adjusted hazard ratio.

Of the 1380 patients with diabetes free of DR and DME at baseline, 414 patients had incident DR or DME at follow-up (ie, 387 cases of DR alone, 16 cases of DME alone, and 11 cases of DR combined with DME). Separate analyses for incident DME (n=27) and VTDR (n=28; including 1 case of severe NPDR) as distinct outcomes were not performed due to the small number of events. The associations of LAP, VAI, and CVAI with incident DR remained unchanged when new-onset DME was added to the outcome of interest or when patients who had new-onset DR within the first year of follow-up were excluded. The strength of associations between visceral obesity indices and incident DR tended to be somewhat attenuated in Cox models without adjustment for BMI; however, the results remained significant (Figure 3).

Sensitivity analysis 1 was performed with time-dependent Cox regression models (n=1380), in which the outcome of interest was redefined as new-onset DR or DME, with models adjusted for sex, age, duration of diabetes, education level, current smoking, regular drinking, BMI, BP, HbA_1c_, serum cholesterol level, eGFR, and use of insulin. Sensitivity analysis 2 was performed with multivariable-adjusted, time-dependent Cox regression models (n=1320), in which patients who had incident DR within the first year of follow-up were excluded. Sensitivity analysis 3 was performed with multivariable-adjusted, time-dependent Cox regression models (N=1403), in which BMI was not adjusted for in the analysis.

The multivariable-adjusted hazard of incident DR at follow-up associated with LAP, VAI, and CVAI remained significant when WC was incorporated as a covariate in the time-dependent Cox model, as well as in the time-fixed Cox model in which baseline values of visceral obesity indices were used (Table S3 in Multimedia Appendix 1).

## Discussion

### Principal Findings

In this prospective cohort study conducted in southern China, we demonstrated longitudinal associations between 3 novel indices of visceral obesity and the incidence of DR in patients with diabetes. During a median follow-up of over 2 years, elevated levels of LAP, VAI, and CVAI were all independently associated with increased risk for new-onset DR, with positive dose-response trends. Our data suggested that visceral obesity was a significant risk factor for DR, with the associations independent of generalized and abdominal obesity. Multivariable-adjusted, time-dependent Cox regression models showed no evidence of effect modification by sex, age, duration of diabetes, BMI, and the presence of concurrent hypertension, dyslipidemia, and decreased renal function.

### Comparison With Existing Literature

A recent systematic review of population-based cohort studies reported an annual incidence of DR ranging from 2.2% to 12.7% worldwide [58], while this study showed a slightly higher incidence rate. The differences might be explained by heterogeneity in the use of 7-field color stereoscopic fundus photographs for DR detection and in patient characteristics (eg, in terms of duration of diabetes) across studies. Over one-third of patients in this study had diabetes for more than 10 years and might be at a more advanced stage of diabetes with higher risks for DR.

The association between obesity and DR has been previously reported in a large number of studies, albeit with equivocal findings [19-21]. The lack of consensus may be due to the failure to address the mutually confounding effect resulting from the strong interrelation between generalized and abdominal obesity [27]. Obesity measures that account for the distribution of adipose tissue, and more specifically, visceral adiposity, have been considered a potentially stronger indicator for the risk of DR [25]. Positive associations between visceral obesity and DR have been reported in clinic-based studies among both Japanese and Singaporean adults with type 2 diabetes [59,60]. In contrast, the community-based Jogjakarta Eye Diabetic Study [61] reported opposite findings from Indonesian adults with type 2 diabetes, while a null association between visceral obesity and DR was observed in French patients with diabetes [62]. Plausible explanations for contradictory results include the use of different measurement methods (eg, CT [59], MRI [62], and BIA [60,61]), racial or ethnic disparities, and studies with relatively small sample sizes.

Simple and validated surrogate indices for measuring VAT, such as LAP [34,50], VAI [35], and CVAI [36], have been proposed given the resource availability and time constraints in routine clinical settings. A cross-sectional study in northern China reported positive associations of LAP and CVAI with DR among adults with type 2 diabetes [40], whereas another study with a similar design showed an inverse association between LAP and DR [39]. Cross-sectional community-based findings in eastern China, however, indicated nonsignificant associations of LAP, VAI, and CVAI with DR [41]. A lack of significant associations between visceral obesity indices and DR was also reported from a northern Chinese cohort of patients with type 2 diabetes [63], based on a time-fixed Cox model in which information on the duration of diabetes was not collected and the number of outcome events of DR as assessed by 4-field fundus photographs was much smaller (ie, 90 vs 406 cases) than that in this study, which took into account the time-varying effect in the Cox model with DR assessed by color stereoscopic fundus photographs of 7-standard fields.

To date, few studies have characterized the dose-response relationship between visceral obesity indices and DR using RCS functions. We found consistent associations of LAP, VAI, and CVAI with risk for DR, which appeared to indicate the presence of dose-response. Given that the shape of the RCS curve is largely influenced by the location and number of knots, the exploration of a specific threshold per se was not the focus of this study. We observed a nonlinear dose-response curve for LAP and VAI, but not for CVAI. This may be in part due to the inclusion of age as a component in the calculation of CVAI [36], yet further research is needed to better understand how different components used for the calculation of these surrogate indices are interacted with in basic molecular mechanisms and key pathogenic processes that drive abnormalities and lesions in the diabetic retina [64].

To our knowledge, this was one of the first studies using a prospective design to investigate the longitudinal associations of LAP, VAI, and CVAI with new-onset DR simultaneously while evaluating potential nonlinear associations. The anthropometric measurements, laboratory tests, and dilated-pupil retinal ophthalmoscopic examinations were performed annually by a regularly trained team of clinical staff who followed standard operating procedures with quality control. We adopted Early Treatment Diabetic Retinopathy Study 7-field color stereoscopic fundus photography, which has long been the gold standard for DR detection [12], to ensure the absence of DR in patients at baseline enrollment and the accurate capture of DR events during follow-up. The use of time-varying measures of visceral obesity indices and covariates in the Cox models took into account the plausible effects of time-varying exposure over the study period. The analyses were systematically performed using the 3 visceral obesity indices as continuous variables and in tertiles, with a consistent methodology adopted to deal with residual confounding and reverse causality. An extensive range of sensitivity analyses based on time-dependent and time-fixed multivariable-adjusted Cox models yielded little difference in estimated associations between visceral obesity indices and the incidence of DR, suggesting the robustness of our findings.

### Underlying Biological Mechanisms

Although the biological mechanisms underlying the association between visceral obesity and DR are not yet fully understood, several hypotheses have been proposed. First, it was found that VAT adipocytes are more metabolically active and have greater lipolytic activity compared to SAT adipocytes [65]. Visceral fat accumulation is associated with a greater tendency to hyperglycemia, hyperinsulinemia, hypertriglyceridemia, and increased apolipoproteins B-rich lipoproteins, all of which could play a role in diabetes-related microvascular complications [65,66]. Second, evidence showed that VAT is more infiltrated with inflammatory cells (macrophages) than SAT [67] and is more capable of generating proinflammatory cytokines such as tumor necrosis factor-α, C-reactive protein, and interleukin-6 [65], which are involved in the pathological mechanisms leading to vascular dysfunction [24]. In addition, the plasminogen activator inhibitor-1, which is expressed more in VAT than SAT, has also been proven to be associated with increased susceptibility to DR [68,69]. Third, elevated vascular endothelial growth factor concentrations associated with visceral fat accumulation have been shown to be a potent mediator of angiogenesis and vascular permeability [11] and play a pivotal role in the retinal microvascular complications of diabetes [70,71]. Taken together, an excess of visceral fat could be linked to a state of chronic systemic inflammation and metabolic abnormalities, thereby predisposing patients to the complex progression of retinal vascular diseases [72].

### Implications for Research and Practice

A recent review concluded that known risk factors for complications of diabetes appear to have limited implications as predictors of retinopathy development or progression [11]. This study provides novel, prospective evidence of the positive association between visceral obesity indices and new-onset DR. Given that stand-alone intensive control of blood glucose and BP might not suffice to significantly reduce DR risk [15], our findings suggest the necessity for regular monitoring of visceral adiposity apart from blood glucose and pressure. As visceral adiposity tends to be ignored by the general public, efforts should be made to increase awareness of the adverse effects of an excess of visceral fat on vascular permeability and growth. This may require tailored health education for high-risk patient groups to improve adherence to healthy lifestyles, thereby reducing excessive visceral fat. This is in line with the most recent American Diabetes Association guideline that advocates lifestyle improvement and obesity management in patients with diabetes [17,73].

From a public health perspective, routine monitoring of visceral obesity indices that are validated and easily implemented in low-resource settings may assist in identifying a broader range of patients who are at risk for DR. This would allow for early detection and timely intervention to avoid irreversible vision loss. As direct access to imaging modalities may not be widely feasible in daily practice [29], alternative approaches using surrogate indices that are broadly applicable to detect and measure intra-abdominal (visceral) fat distribution have gained increasing popularity. This study demonstrates the potential utility of visceral obesity indices, calculated using routinely available demographic, anthropometric, and biochemical measures, in predicting new-onset DR outcomes in remote or rural settings in low- and middle-income countries. Of note, our findings did not indicate which of the 3 indices was superior to one another but rather suggested opportunities for preventing DR through the application of these indices in risk assessment and management. Further interventional studies aimed at examining the effectiveness of continuous monitoring of visceral obesity indices for the prevention of DR are expected to offer important insights into the long-term management of diabetes and inform targeted public health intervention strategies and clinical guidelines.

### Limitations

This study has some limitations that merit consideration. First, the VAT was not directly measured using CT or MRI or estimated using DEXA or BIA due to the resource constraints; instead, sex-specific equation-based indices that were previously shown to be valid and reliable were used [34-36]. Second, the association of visceral obesity indices with incident DR may be underestimated, as the retinopathy outcome may not occur during the study period. Third, nearly one-third of patients enrolled at baseline were lost to follow-up or had missing outcome data. They appeared to be slightly older and had poorer glycemic control compared to their counterparts who attended follow-up, which might undermine the relationship between visceral obesity indices and incident DR in the final analysis despite largely comparable baseline characteristics concerning sex, duration of diabetes, lifestyles, comorbidity, and the majority of clinical measurements. Fourth, a separate analysis for incident DME or VTDR alone was not performed due to the small number of events. Fifth, we cannot rule out the possibility of residual bias because of confounding by unmeasured factors. Last but not least, the generalizability of our findings in Chinese diabetes to other patient populations should be interpreted with caution. Given that the present study was originally centered around evaluating the potential of visceral obesity indices in predicting new-onset DR rather than serving as a comprehensive validation with DEXA or BIA per se, further investigations on the basis of imaging techniques in a larger series of multiethnic patients are warranted to strengthen the evidence for the visceral adiposity-DR relationship.

In conclusion, this study provides prospective evidence that visceral obesity as measured by LAP, VAI, or CVAI is significantly associated with increased risk for new-onset DR, independent of generalized and abdominal obesity in Chinese patients with diabetes. Our findings may suggest the necessity of incorporating regular monitoring of visceral obesity indices in clinical practice to enhance population-based prevention for DR.

## Appendix

Multimedia Appendix 1 Supplemental material.

## Abbreviations

aHR: adjusted hazard ratio
BIA: bioelectrical impedance analysis
BP: blood pressure
CT: computed tomography
CVAI: Chinese visceral adiposity index
DEXA: dual-energy x-ray absorptiometry
DME: diabetic macular edema
DR: diabetic retinopathy
eGFR: estimated glomerular filtration rate
ETDRS: Early Treatment Diabetic Retinopathy Study
HbA1c: hemoglobin A1c
HR: hazard ratio
LAP: lipid accumulation product
MRI: magnetic resonance imaging
NPDR: nonproliferative diabetic retinopathy
RCS: restricted cubic spline
SAT: subcutaneous adipose tissue
VAI: visceral adiposity index
VAT: visceral adipose tissue
VTDR: vision-threatening diabetic retinopathy
WC: waist circumference
WHR: waist-to-hip ratio
WHtR: waist-to-height ratio

## Data Availability

The data sets generated during and/or analyzed during the current study are available from the corresponding author upon reasonable request.
