# Supplementary material for: Association of Visceral Obesity Indices With Incident Diabetic Retinopathy in Patients With Diabetes: Prospective Cohort Study
Source: JMIR Public Health Surveill. 2024 Feb 6;10:e48120. doi: 10.2196/48120 (PMC10879974; doi:10.2196/48120)
Supplement: Multimedia Appendix 1 [file publichealth_v10i1e48120_app1.doc]

**Multimedia Appendix 1**

**Supplementary Methods**

***Inclusion and exclusion criteria***

Eligible participants were: (1) aged 30 to 80 years; (2) patients with a clinical diagnosis of diabetes; (3) having no prior history of ocular treatment; and (4) capable of completing a full ophthalmoscopic exam. Exclusion criteria included: (1) serious systemic diseases such as resistant hypertension, severe cardiovascular and cerebrovascular disease, cancer, or nephritis; (2) type 1 (insulin-dependent) diabetes or gestational diabetes; (3) history of general surgery, thrombolysis, or renal dialysis; (4) glaucoma, vitreous degeneration, or amblyopia; (5) history of retinal surgery, laser treatment of the retina, or intraocular injection; (6) cognitive disorders, mental impairment, or incapable of independent communication with clinical staff; and (7) poor-quality fundus photograph or optical coherence tomography (OCT) image which precluded the assessment of diabetic retinopathy (DR) and diabetic macular oedema (DME).

***Variables***

Education level was categorised as junior secondary school or below, senior secondary school, and college or above. Smoking status was categorised as current smoking (i.e., daily cigarette smoking) and others. Drinking status was categorised as regular drinking and others. Regular drinkers referred to those who frequently engaged in alcohol drinking for more than an equivalent of 25 g (for males) or 15 g (for females) of daily alcohol consumption [45]; or for ≥4 days per week [46]. BMI was categorised as <24 kg/m^2^ and ≥24 kg/m^2^ (including overweight and obesity) [45]. Hypertension was defined as systolic BP ≥140 mmHg and/or diastolic BP ≥90 mmHg on repeated clinical measurements, and/or on antihypertensive medication [47]. Dyslipidaemia was considered present if triglycerides (TG) ≥2.3 mmol/l, and/or total cholesterol (TC) ≥6.2 mmol/l, and/or LDL cholesterol (LDL-C) ≥4.1 mmol/l, and/or HDL cholesterol (HDL-C) <1.0 mmol/l, and/or on lipid-lowering medication [48]. Decreased renal function was determined as estimated glomerular filtration rate (GFR) <90 mL/min per 1.73 m^2^ (including mildly decreased GFR) according to the guideline-recommended target level [49].

***Measurements and calculations***

The sex-specific equations for calculating lipid accumulation product (LAP), visceral adiposity index (VAI), and Chinese visceral adiposity index (CVAI) were as follows.

- Men:

LAP = [WC (cm) – 65] × TG (mmol/L) [50]

VAI = WC (cm) / [39.68 + 1.88 × BMI (kg/m^2^)] × [TG (mmol/L) / 1.03]

× [1.31 / HDL-C (mmol/L)] [35]

CVAI = –267.93 + 0.68 × age (y) + 0.03 × BMI (kg/m^2^) + 4.00 × WC (cm)

+ 22.00 × Log_10_TG (mmol/L) – 16.32 × HDL-C (mmol/L) [36]

- Women:

LAP = [WC (cm) – 58] × TG (mmol/L) [50]

VAI = WC (cm) / [36.58 + 1.89 × BMI (kg/m^2^)] × [TG (mmol/L) / 0.81]

× [1.52 / HDL-C (mmol/L)] [35]

CVAI = –187.32 + 1.71 × age (y) + 4.23 × BMI (kg/m^2^) + 1.12 × WC (cm)

+ 39.76 × Log_10_TG (mmol/L) – 11.66 × HDL-C (mmol/L) [36]

***Model construction***

In the Cox proportional hazard models with time-dependent variables, the entire follow-up time for each patient was divided into different time windows (i.e., the time intervals between repeated follow-up measurements). Separate Cox regression analyses were performed using the specific value of the time-dependent variable at the beginning of that specific time window. The weighted average of all the time window-specific hazard ratios (HR) was calculated and presented as the result of the analysis [51], with or without adjustment for sex, age, duration of diabetes, education level, current smoking, regular drinking, body mass index, blood pressure, haemoglobin A1c, serum cholesterol level, estimated glomerular filtration rate, and use of insulin.

**Figure S1 Study flow chart**


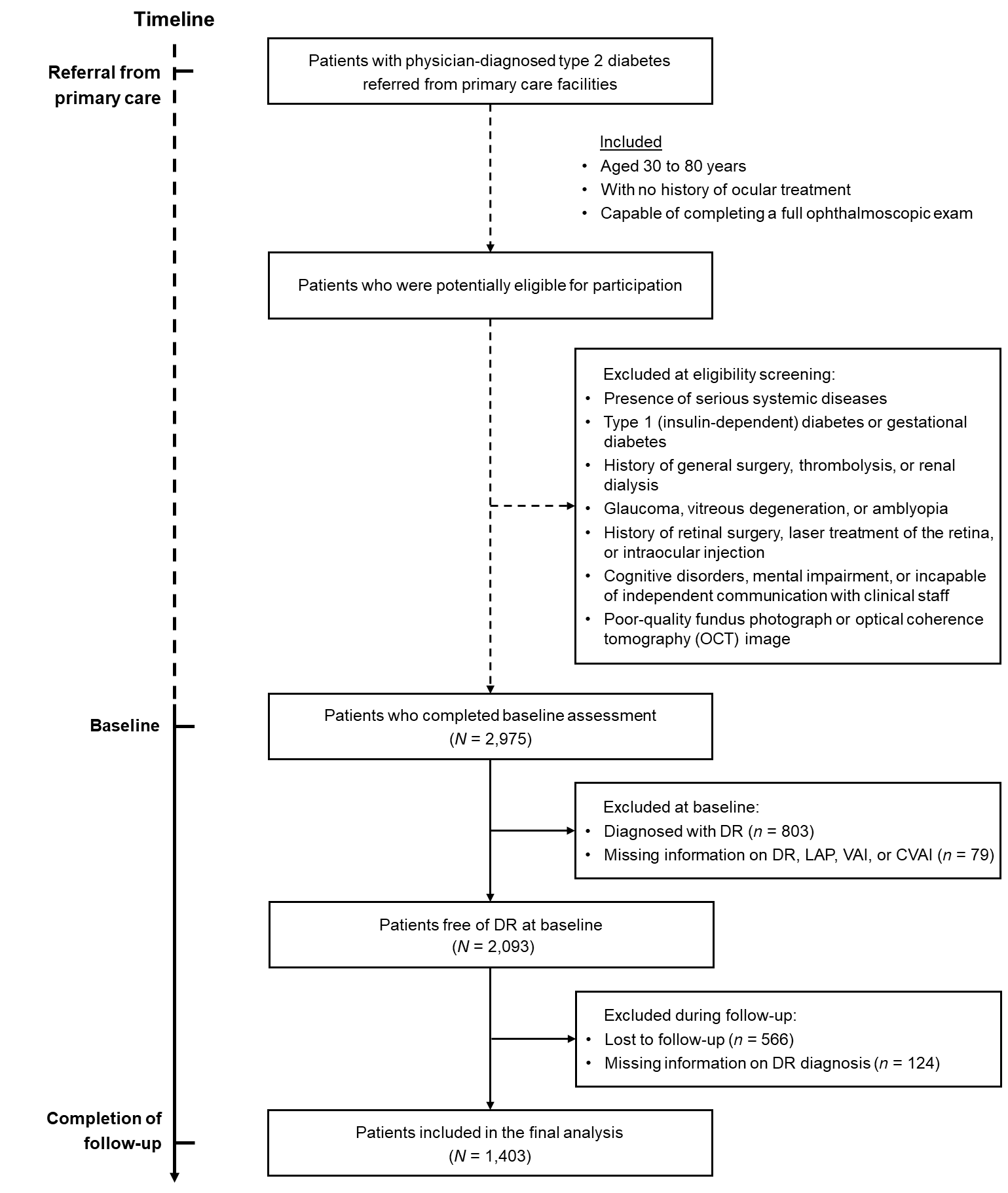


Note: DR, diabetic retinopathy; LAP, lipid accumulation product; VAI, visceral adiposity index; CVAI, Chinese visceral adiposity index.

**Table S1 Correlation matrix of obesity measurements**

|  | **LAP** | **VAI** | **CVAI** | **BMI** | **WC** | **WHtR** | **WHR** |
| --- | --- | --- | --- | --- | --- | --- | --- |
| LAP | 1.000 |  |  |  |  |  |  |
| VAI | 0.845 | 1.000 |  |  |  |  |  |
| CVAI | 0.635 | 0.375 | 1.000 |  |  |  |  |
| BMI | 0.432 | 0.163 | 0.753 | 1.000 |  |  |  |
| WC | 0.492 | 0.172 | 0.867 | 0.753 | 1.000 |  |  |
| WHtR | 0.483 | 0.197 | 0.857 | 0.763 | 0.883 | 1.000 |  |
| WHR | 0.320 | 0.131 | 0.614 | 0.284 | 0.736 | 0.676 | 1.000 |

Note: LAP, lipid accumulation product; VAI, visceral adiposity index; CVAI, Chinese visceral adiposity index; BMI, body mass index; WC, waist circumference; WHtR, waist-to-height ratio; WHR, waist-to-hip ratio.

Pearson’s correlation coefficient (*r*) was presented. All *P* values <0.001.

**Table S2** **Baseline characteristics of patients excluded during follow-up and those included in the final analysis**

| **Characteristics** | **Patients excluded during follow-up** | **Patients included in the final analysis** | ***P* value** |
| --- | --- | --- | --- |
|  | **(n = 690)** | **(N = 1,403)** |  |
| Women, % | 426 (61.7) | 816 (58.2) | 0.117 |
| Age, years | 65.23 (8.37) | 64.45 (7.59) | 0.038 |
| ≥65 years, % | 381 (55.2) | 701 (50.0) | 0.024 |
| Duration of diabetes, years | 5.7 (2.7-10.1) | 6.5 (3.0-11.0) | 0.055 |
| ≥10 years, % | 214 (31.0) | 506 (36.1) | 0.030 |
| Education level |  |  |  |
| Junior secondary school or below, % | 266 (38.5) | 436 (31.1) | <0.001 |
| Senior secondary school, % | 306 (44.3) | 574 (40.9) |  |
| College or above, % | 118 (17.1) | 393 (28.0) |  |
| Current smoking, % | 113 (16.4) | 185 (13.2) | 0.104 |
| Regular drinking, % | 78 (11.3) | 132 (9.4) | 0.247 |
| Use of insulin, % | 83 (12.0) | 233 (16.6) | 0.010 |
| Presence of comorbidity |  |  |  |
| Hypertension, % | 374 (54.2) | 794 (56.6) | 0.301 |
| Dyslipidaemia, % | 451 (65.4) | 938 (66.9) | 0.496 |
| Decreased renal function, % | 282 (40.9) | 577 (41.1) | 0.911 |
| BMI, kg/m² | 24.69 (3.52) | 24.63 (3.27) | 0.707 |
| ≥24 kg/m², % | 406 (58.8) | 781 (55.7) | 0.168 |
| WC, cm | 87.84 (9.50) | 85.95 (9.12) | <0.001 |
| SBP, mmHg | 133.44 (18.72) | 132.83 (18.12) | 0.477 |
| DBP, mmHg | 70.23 (10.84) | 70.31 (10.23) | 0.884 |
| HbA1c, % | 7.04 (1.42) | 6.81 (1.22) | <0.001 |
| TC, mmol/L | 4.92 (1.12) | 4.82 (1.06) | 0.043 |
| TG, mmol/L | 2.09 (1.41-3.02) | 1.90 (1.34-2.86) | 0.014 |
| LDL-C, mmol/L | 3.06 (1.00) | 3.03 (0.95) | 0.487 |
| HDL-C, mmol/L | 1.28 (0.40) | 1.30 (0.41) | 0.391 |
| SCr, μmol/L | 70.28 (21.48) | 71.37 (19.67) | 0.264 |
| eGFR, mL/min per 1.73 m^2^ | 90.44 (17.70) | 90.30 (16.91) | 0.860 |
| LAP | 53.32 (31.74-82.50) | 48.00 (29.12-77.22) | 0.039 |
| VAI | 2.72 (1.62-4.48) | 2.69 (1.54-4.43) | 0.460 |
| CVAI | 123.21 (99.17-145.30) | 118.18 (94.77-141.17) | 0.016 |

Note: BMI, body mass index; WC, waist circumference; SBP, systolic blood pressure; DBP, diastolic blood pressure; HbA1c, haemoglobin A1c; TC, total cholesterol; TG, triglycerides; LDL-C, low-density lipoprotein cholesterol; HDL-C, high-density lipoprotein cholesterol; SCr, serum creatinine; eGFR, estimated glomerular filtration rate; LAP, lipid accumulation product; VAI, visceral adiposity index; CVAI, Chinese visceral adiposity index.

Data were shown as mean (standard deviation) or median (interquartile range) according to the distribution of observations for continuous variables, and *n* (%) for categorical variables. The two-sample *t*-test, Mann-Whitney U test, or the chi-square test, where appropriate, was used for between-group comparison.

**Table S3 Associations of LAP, VAI, and CVAI with incident DR in** sensitivity analyses

| **Visceral obesity indices** | **Sensitivity analysis 1** | | **Sensitivity analysis 2** | | **Sensitivity analysis 3** | |
| --- | --- | --- | --- | --- | --- | --- |
|  | **aHR (95% CI)** | ***P* value** | **aHR (95% CI)** | ***P* value** | **aHR (95% CI)** | ***P* value** |
| **LAP** |  |  |  |  |  |  |
| Per SD increase | 1.14 (1.02-1.29) | 0.027 | 1.27 (1.10-1.46) | 0.001 | 1.25 (1.09-1.43) | 0.001 |
| Tertile 1 | 1.00 [Ref] |  | 1.00 [Ref] |  | 1.00 [Ref] |  |
| Tertile 2 | 1.22 (0.90-1.64) | 0.198 | 1.40 (0.99-1.98) | 0.058 | 1.27 (0.90-1.78) | 0.174 |
| Tertile 3 | 1.51 (1.10-2.07) | 0.011 | 2.00 (1.37-2.92) | <0.001 | 1.72 (1.20-2.47) | 0.003 |
| *P* for trend | 0.011 |  | <0.001 |  | 0.003 |  |
| **VAI** |  |  |  |  |  |  |
| Per SD increase | 1.16 (1.05-1.29) | 0.003 | 1.23 (1.10-1.37) | <0.001 | 1.24 (1.11-1.39) | <0.001 |
| Tertile 1 | 1.00 [Ref] |  | 1.00 [Ref] |  | 1.00 [Ref] |  |
| Tertile 2 | 1.58 (1.18-2.13) | 0.002 | 1.59 (1.14-2.21) | 0.006 | 1.65 (1.19-2.29) | 0.002 |
| Tertile 3 | 1.76 (1.31-2.37) | <0.001 | 1.91 (1.37-2.65) | <0.001 | 1.84 (1.33-2.55) | <0.001 |
| *P* for trend | 0.001 |  | <0.001 |  | 0.001 |  |
| **CVAI** |  |  |  |  |  |  |
| Per SD increase | 1.26 (1.04-1.54) | 0.021 | 1.73 (1.31-2.30) | <0.001 | 1.79 (1.33-2.40) | <0.001 |
| Tertile 1 | 1.00 [Ref] |  | 1.00 [Ref] |  | 1.00 [Ref] |  |
| Tertile 2 | 1.26 (0.91-1.73) | 0.160 | 1.47 (1.01-2.13) | 0.044 | 1.48 (1.02-2.15) | 0.040 |
| Tertile 3 | 1.41 (0.94-2.12) | 0.094 | 1.89 (1.15-3.12) | 0.013 | 2.16 (1.30-3.59) | 0.003 |
| *P* for trend | 0.150 |  | 0.013 |  | 0.006 |  |

Note: LAP, lipid accumulation product; VAI, visceral adiposity index; CVAI, Chinese visceral adiposity index; aHR, adjusted hazard ratio; CI, confidence interval; SD, standard deviation; Ref, reference.

Sensitivity analysis 1 was performed with time-fixed Cox regression models in which baseline measurements of LAP, VAI, and CVAI were used, with adjustments for sex, age, duration of diabetes, education level, current smoking, regular drinking, body mass index, blood pressure, haemoglobin A1c, serum cholesterol level, estimated glomerular filtration rate, and use of insulin. Sensitivity analysis 2 was performed with multivariable-adjusted, time-dependent Cox regression models in which waist circumference (WC) was incorporated as a covariate. Sensitivity analysis 3 was performed with multivariable-adjusted, time-fixed Cox regression models in which WC was incorporated as a covariate. 95% confidence interval in parentheses.
